# Effect of environmental stressors on the mRNA expression of ecdysone cascade genes in *Chironomus riparius*

**DOI:** 10.1007/s11356-021-16339-3

**Published:** 2021-09-13

**Authors:** Mercedes de la Fuente, Raquel Martín Folgar, Pedro Martínez-Paz, Estrella Cortés, José Luis Martínez-Guitarte, Mónica Morales

**Affiliations:** 1 Grupo de Biología y Toxicología Ambiental, Facultad de Ciencias, Universidad Nacional de Educación a Distancia, UNED. Urbanización Monte Rozas. Avda. Esparta s/n. Ctra. de Las Rozas al Escorial Km. 5, 28232 Las Rozas–Madrid, Spain; 2 Grupo de Biomedicina en Cuidados Críticos, Facultad de Medicina, Universidad de Valladolid, Av. Ramón y Cajal 7, 47005 Valladolid, Spain

**Keywords:** Endocrine-disrupting chemicals (EDCs), *Chironomus riparius*, Toxicity, Transcriptional response

## Abstract

**Abstract:**

Chemical compounds produced by humans are continuously reaching the environment. In this work, we characterised the expression patterns of important endocrine-related genes involved in the ecdysone pathway in the fourth larval instar of the model species *Chironomus ripariu*s after exposure to three chemicals: ethinyl oestradiol (EE), nonylphenol (NP) and bis(tributyltin) oxide (TBTO). We used real-time PCR to analyse the gene expression levels of *ecdysone receptor* (*EcR*) and *ultraspiracle* (*usp*), two genes that encode the dimerising partners of the functional ecdysone receptor; the orphan receptor *ERR* (*oestrogen-related receptor*), with an unknown function in invertebrates; and *E74*, an early response gene induced by ecdysteroids. We estimated the bioaccumulation potential, bioavailability and physicochemical properties of these chemicals, together with a number of other exogenous agents known to interfere with the hormonal system. We also provide a review of previous transcriptional studies showing the effect of all these chemicals on ecdysone cascade genes. This analysis provides useful data for future ecotoxicological studies involving invertebrate species.

**Capsule:**

Changes in transcriptional activities of *EcR*, *E74*, *usp* and *ERR* genes after exposure to endocrine-disrupting chemicals would be useful as molecular bioindicators of endocrine disruption in *Chironomus ripariu*s.

## Introduction

Endocrine-disrupting chemicals (EDCs) are substances that alter a wide variety of physiological, biochemical and/or molecular processes in an organism by interfering with the regulatory networks of endogenous hormones. The impacts of the disruption are quite diverse and involve development, growth, reproduction, neurological and immune processes (Cuvillier-Hot and Lenoir, 2020). Beyond the influences that these effects have on worldwide biodiversity, the characterisation of EDC responses in invertebrates is tremendously interesting and widely recognised among environmental toxicologists due to the central role of invertebrate species in ecosystems and, consequently, to their importance in ecotoxicological testing and for monitoring environmental conditions. Toxicity testing provides information about the physiological effects of toxicants, but there is still a gap with regard to the mechanisms of action of the toxicant and the response of the organism at the cellular and molecular levels. In this sense, the changes in transcriptional activities of several selected genes after exposure to EDCs have proven to be a very useful tool as molecular bioindicators of endocrine disruption in aquatic invertebrates (Kim et al., 2015; Poynton and Vulpe, 2009). *Chironomus riparius* is considered a convenient and suitable model organism for these ecotoxicological assessments. The United States Environmental Protection Agency (US EPA), Organization for Economic Co-operation and Development (OECD) and the American Society for Materials Testing (ASTM) have published standardised protocols for the development of toxicity tests with these insects (ASTM, 2006; OECD, 2004, 2010, 2011). Accordingly, myriad studies have been performed to distinguish the effects of different environmental pollutants on the expression of genes of interest in this insect (Aquilino et al., 2016; Herrero et al., 2017, 2018; Martínez-Paz et al., 2012, 2017; Morales et al., 2013, 2014, 2020; Nair and Choi, 2012; Nair et al., 2013; Ozáez et al., 2013, 2014; Park and Kwak, 2010; Planelló et al., 2007, 2008, 2010, 2011, 2015).

It is essential to have accumulated knowledge regarding the putative mode of action of EDCs to choose the best biomarkers, to standardise the transcriptional studies for toxicity tests and to allow the extrapolation of the observed effects to wildlife. Different molecular regulatory pathways have been revealed to be susceptible to endocrine disruption, some of which are specific to invertebrates. In *C. riparius*, one of the primary hormonal pathways susceptible to alteration is that triggered by 20-hydroxiecdysone (20E) (Cuvillier-Hot and Lenoir, 2020), one of the main steroid hormones in insects. Previous studies carried out in salivary glands exposed to 20E under a variety of conditions demonstrated the molecular basis of 20E action in *Drosophila melanogaster*. In the early 1970s, Ashburner (1974) proposed that 20E binds to the ecdysone receptor (EcR), which is the ecdysone receptor/ultraspiracle (EcR/USP) heterodimer (Koelle et al., 1991; Lezzi et al., 1999; Yao et al., 1993). The 20E receptor complex differentially regulates a multitiered hierarchy of responses involving several classes of ‘early’ and ‘late’ genes, so that it activates the expression of the early genes (whereas a receptor without ligand acts as a repressor) and represses the late ones. The protein products of the early genes are transcription factors that derepress the expression of the late gene transcription and, concomitantly, repress their own expression. The late genes encode the proteins responsible for the temporal and tissue-specific responses in target tissues (Dhadialla et al., 2005; Karim and Thummel, 1992; Nakagawa and Henrich, 2009; Uyehara and McKay, 2019). The wide array of assays performed in *C. riparius* exposed to several chemicals, referenced above, have analysed the up/downregulation of some genes belonging to this so-called ecdysone cascade – most of them involve *EcR*, *usp* and *E74* genes, as well as oestrogen-related receptor (*ERR*), whose function is not fully understood but whose activation seems very relevant during developmental stages (Bunce and Campbell, 2010).

Despite the accumulated information, additional work is required to ascertain the effect of each pollutant. This work requires molecular data combined with other aspects such as bioaccumulation and elimination of the compounds. The aim of this work is to combine the data obtained for transcriptional activity, from the literature and for additional toxicants studied here, with the properties of the compounds to provide a broader view of the EDCs (see Supplementary information) as agonists/antagonists of hormonal receptors.

## Materials and methods

### Animals and treatments

Fourth instar *C. riparius* larvae served as the experimental model organism. Traditionally, the studies for the characterisation of the different selected compounds will focus on the case of *Chironomus* in aquatic larvae, fundamentally in the fourth stage since it is the stage where mortality and delay in emergence have been considered of maximum impact to prevent effects at the population level. Moreover, according to previous studies, younger individuals may be more sensitive to toxicity caused by exposure to certain compounds. They were maintained under constant aeration at 19°C ± 1°C and under 16:8 h light-dark photoperiods for several generations according to toxicity testing guidelines (OECD-EPS, TG18). Larvae were maintained in culture medium (0.5 mM CaCl_2_, 1 mM NaCl, 1 mM MgSO_4_, 0.1 mM NaHCO_3_, 0.025 mM KH_2_PO_4_, 0.01 mM FeCl_3_) supplemented with nettle leaves, fish feed and cellulose paper. The larvae were exposed to the chemical compounds diluted in culture medium for 24 h and 96 h with constant aeration at 19°C ± 1°C (Martínez-Paz et al., 2012). We selected doses based on previous experiments in our group (Martínez-Paz et al., 2013, 2014, 2017) and in other organisms and cell cultures and concentrations detected in the environment (Dann and Hontela, 2011; Hinther et al., 2011; Perron et al., 2012). Fourth instar larvae were submitted to 0.05, 0.5 and 5 mg/L ethinyl estradiol (EE) (Sigma-Aldrich); 1, 10 and 100 μg/L p-nonylphenol (NP) (Sigma-Aldrich); and 0.1, 1, 10, 100 and 1000 ng/L bis(tributyltin) oxide (TBTO) (Sigma-Aldrich). Each treatment comprised three replicates, and there were three independent experiments in each analysis, using larvae arising from six different egg masses (same age or days after hatching). We used three glasses per replica, concentration and xenobiotic. The experimental units (*n* = 3) consisted of glass flasks (5.5 cm in diameter) containing 50 ml of the experimental culture medium (with or without chemical compound). Ten fourth instar *C. riparius* larvae were added to each experimental unit. The control larvae used in each case were exposed to the same concentration of solvent (ethanol 0.02%) as the corresponding treatment and were also measured in triplicate. After the exposure, larvae were stored at −80°C until RNA isolation was carried out.

### RNA isolation and reverse transcription

We isolated RNA and generated complementary DNA (cDNA) by reverse transcription following the procedures of previous works (Martínez-Paz et al., 2012, 2014, 2017; Morales et al., 2013, 2014).

### Real-time PCR

We used cDNA as the template for real-time PCR to analyse the mRNA expression profile of *EcR*, *E74*, *usp* and *ERR* genes in control and treated samples. The sequences of all gene-specific primers used in this study are indicated in Table 1. We used *β-actin*, *Glyceraldehyde 3-phosphate dehydrogenase* (*GAPDH*) and *ribosomal protein L13* (*rpL13*) as reference genes to normalize the transcript expression of target genes (Martínez-Guitarte et al., 2007). The reference genes presented a coefficient of variation <0.25 and an *M*-value <0.5 (Hellemans et al., 2007). We used real-time PCR conditions from a previous study (Martínez-Paz et al., 2017). The software used was CFX Maestro qPCR Analysis.

**Table 1:** Primers used for real-time RT-PCR of genes from *Chironomus riparius*

Gene	Oligo name	Amplification efficiency	Amplicon length	Primer DNA sequence	GenBank accession n°
*β-actin*	Actin F	104.0%	201 bp	5´-GATGAAGATCCTCACCGAACG-3´	KA184592
	Actin 2R			5´-CGGAAACGTTCATTACCG-3´	
*GAPDH*	GAPDH F	96.6%	110 bp	5´-GGTATTTCATTGAATGATCACTTTG-3´	EU999991
	GAPDH R			5´-TAATCCTTGGATTGCATGTACTTG-3´	
*rpL13*	L13 F	109.3%	351 bp	5´-AAGCTGCTTTCCCAAGAC-3´	EF179386
	L13 R			5´-TTGGCATAATTGGTCCAG-3´	
*EcR*	EcR rt F	106.6%	180 bp	5´-CCATCGTCATCTTCTCAG-3´	KJ135024
	EcR rt R			5´-TGCCCATTGTTCGTAG-3´	
*usp*	USP F	108.1%	114 bp	5´-GCCCAATCATCCGTTAAGTGG-3´	HP608040
	USP R			5´-CGTTTGAAGAATCCTTTACATCC-3´	
*E74*	E74 F	103.2%	111 bp	5´-TCTTACTGAAACTTCTTCAAGATCG-3´	KA177910.1
	E74 R			5´-GCTTTGAGACAGCTTTGGAATCG-3´	
*ERR*	ERR F	104.7%	222 bp	5´-CTCAGCAAGTAAGGAGGAG-3´	GU070740
	ERR R			5´-CGTCTAATAATGTGATCGG-3´	

### Statistical analysis

We compared between the control and the treated larvae using analysis of variance (ANOVA) with Dunnett’s multiple comparison tests. We normalised *EcR*, *E74*, *usp* and *ERR* mRNA levels against the expression of the reference genes (*β-actin*, *GAPDH* and *rpL13*) in the same samples. We used SPSS 22.0 software (IBM) for statistical analysis. We checked the data for normal distribution and variance homogeneity using the Kolmogorov–Smirnov test and Levene’s test, respectively. We considered a difference to be significant at *p* < 0.05.

### Molecular descriptors, principal component analysis and hierarchical clustering analysis

We calculated the molecular weight and volume, as well as other molecular structure indicators, solvation properties (such as the aqueous solubility, [log *S*] and the octanol-water partition coefficient [log *P*]) and the bioconcentration factor (BCF) using the following software: molinspiration (Molinspiration Cheminformatics free web services, n.d.) EPI Suite (US EPA, 2020) and eDragon (http://www.vcclab.org/lab/edragon/; Tetko et al., 2005). We analysed the estimated values with principal component analysis (PCA) and hierarchical clustering analysis (HCA), using ClustVis (Metsalu and Vilo, 2015; https://biit.cs.ut.ee/clustvis/).

## Results and discussion

### Transcriptional response of the EcR gene under different exposures to chemical compounds

**Fig. 1:**
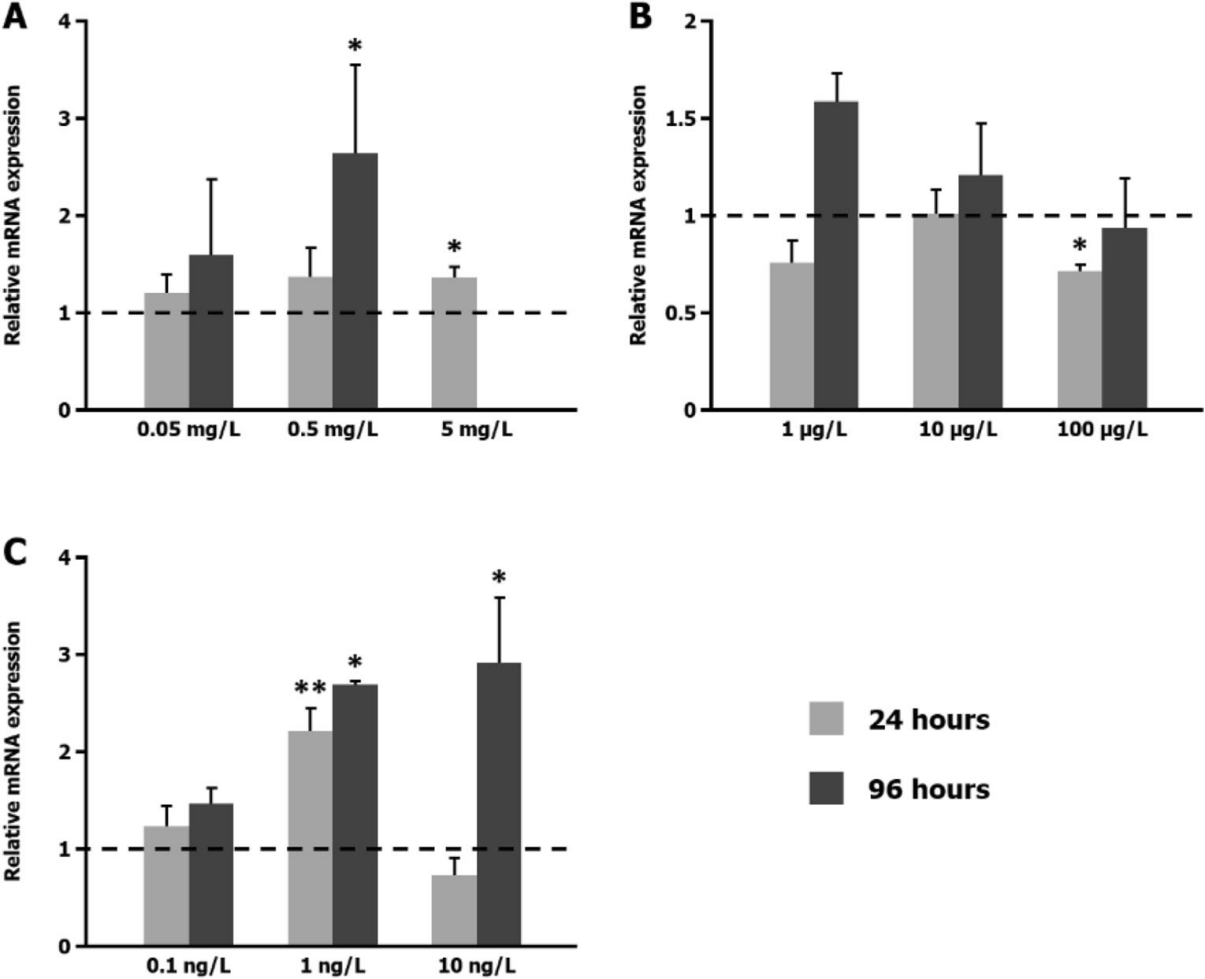
Effect of ethinyl oestradiol (**A**), p-nonylphenol (**B**) and bis(tributyltin) oxide (**C**) exposure on *EcR* gene expression. The graphs present the relative mRNA levels, measured by real-time PCR with primers and reference genes indicated in ‘Real-time PCR’, after 24 h and 96 h exposure, relative to untreated control larvae, for which the expression level was set to 1

We examined the effects of 24 h and 96 h EE, NP and TBTO exposure on *EcR* expression in *C. riparius* fourth instar larvae using real-time PCR; 24 h exposure to 5 mg/L EE and 96 h exposure to 0.5 mg/L EE increased *EcR* mRNA levels (Fig. 1A). Exposure to 5 mg/L EE for 96 h was lethal for the larvae, so there is no data with regard to *EcR* expression. There was a significant decrease in *EcR* expression in larvae exposed to 100 μg/L NP for 24 h (Fig. 1B). However, mRNA levels were increased after 96 h exposure to 1 and 10 μg/L NP (Fig. 1B). TBTO exposure increased *EcR* expression, doubling the levels found in control larvae. The effect was evident at 24 h for 1 ng/L TBTO and at 96 h for 1 and 10 ng/L TBTO (Fig. 1C).

The impact of several pollutants in the transcriptional activity of *EcR* has been widely studied (Herrero et al., 2015, 2017, 2018; Martínez-Paz et al., 2012, 2017; Morales et al., 2013, 2014, 2020; Nair and Choi, 2012; Nair et al., 2013; Ozáez et al., 2013, 2014; Planelló et al., 2007, 2008, 2010, 2011, 2015; Xie et al., 2019). This gene encodes a key regulator in the ecdysone-inducible gene activation cascade in insects (Huet et al., 1995; Shirai et al., 2012). Its responsiveness makes it particularly relevant in toxicological studies, and it is considered a suitable environmental biomarker. Exposure to most of the organic compounds that have been studied causes an early upregulation of the *EcR* gene, mimicking the response of this gene to the ecdysteroid hormone 20E. Moreover, studies performed at different times with fourth instar larvae treated with the fungicide vinclozolin (VZ) have shown *EcR* upregulation at 24 h, followed by a return to control levels at 48 h (Aquilino et al., 2016). This sequential behaviour is similar to that observed in the presence of 20E (Ozáez et al., 2014). The main chemicals that do not induce the aforementioned effect include di(2-ethylhexyl) phthalate (DEHP) and butyl benzyl phthalate (BBP) (Herrero et al., 2015, 2017) – both causing *EcR* downregulation at longer exposure times; and bisphenol F (BPF), benzophenone-3 (BP-3), 4-hidroxybenzophenone (4-HB), octocrylene (OC) and DEHP – all of which do not alter *EcR* expression after short-term treatment (24 h) (see Supplementary information). All these compounds are proven endocrinal disruptors, but according to these results, they do not mimic the 20E effects.

The results of the test performed with the oestrogen analogue EE (Fig. 1A) and with the organometallic compound TBTO (Fig. 1C) allow us to add them to the group of compounds that upregulate *EcR*. The gene is upregulated from either short- or long-term exposure, revealing a different mode of action of this substance relative to VZ and 20E (see Supplementary information).

**Fig. 2:**
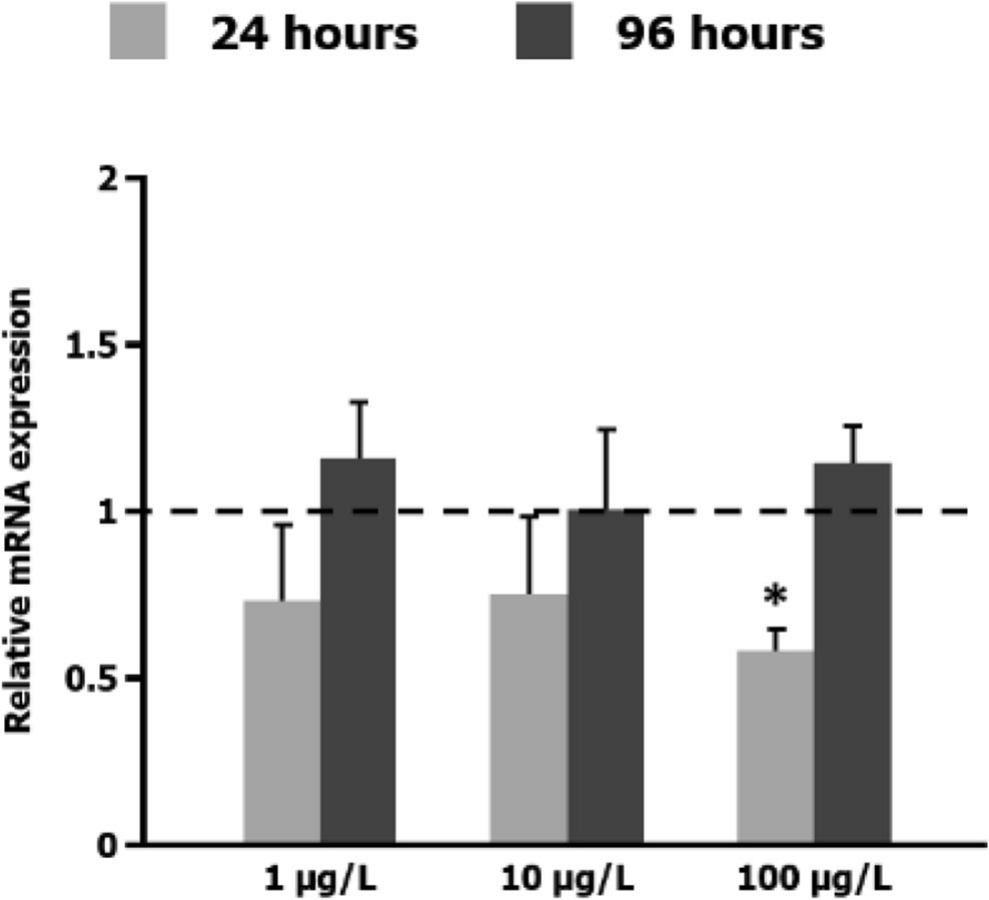
Effect of p-nonylphenol exposure on *ERR* gene expression. The graph presents the relative *ERR* mRNA expression, measured by real-time PCR with primers and reference genes indicated in ‘Real-time PCR’, after 24 h and 96 h exposure, relative to untreated control larvae for which the expression level was set to 1

On the contrary, NP upregulated *EcR* at the lowest dosage and the longer exposure time (Fig. 1B), showing a dose-dependent behaviour inverse to that of EE and TBTO. The *EcR* was significantly increased upon exposure to different concentrations of NP (0.01 and 0.05 mg/L) in a dose- and time-dependent manner (Nair and Choi, 2012). Testing a wider range of concentrations, an inverse relation between dose and expression became apparent: This gene was even downregulated at a higher NP dose (0.1 mg/L) and a shorter exposure time (24 h). By contrast, previous studies revealed a significant increase in *ERR* mRNA expression in *C. riparius* larvae exposed to the same dosages (1, 10 and 100 μg/L) of NP for 24 h and 96 h (Park and Kwak, 2010). To confirm the different behaviour of *EcR* and *ERR* expression in the presence of NP, we carried out a new set of experiments with the same conditions that we used to evaluate *EcR* expression. There was no *ERR* upregulation, as had been previously reported. On the contrary, exposure to 100 μg/L NP for 24 h inhibited *ERR* expression (Fig. 2). The response was quite similar to that found for *EcR*; this finding could suggest some kind of relationship between both receptors in response to NP.

In summary, based on these results, there are different patterns of response when *EcR* is analysed, suggesting that there is a different underlying molecular mechanism of toxicity for the studied EDCs. The importance of the kinetics of the response at the molecular level is highlighted by the present results. Hence, future work should assess the effect at different treatment times and at several sub-lethal dosages, an endeavour that could help to identify the specific pattern of changes for each compound in relation to *EcR* transcription.

### Transcriptional response of other ecdysone pathway genes after long-term TBTO exposure

**Fig. 3:**
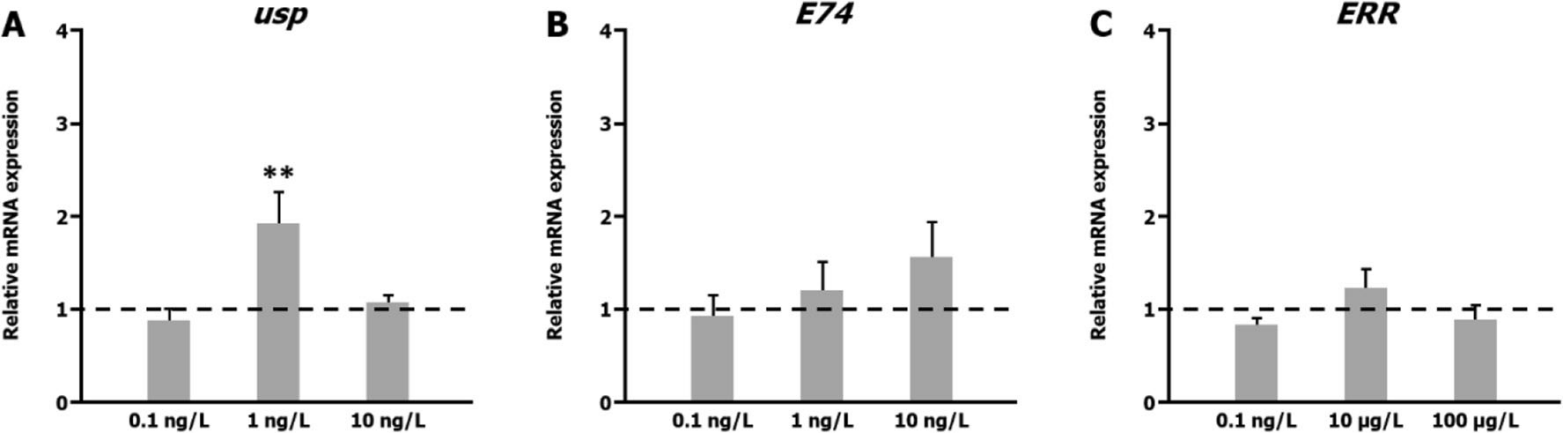
Effect of bis(tributyltin) oxide (C) exposure on *usp*, *E74* and *ERR* gene expression. The graphs present the relative *usp* (**A**), *E74* (**B**) and *ERR* (**C**) mRNA levels, measured by real-time PCR with primers and reference genes indicated in ‘Real-time PCR’, after 96 h exposure to TBTO, relative to untreated control larvae, for which the expression level was set to 1

Steroid hormones exert their effects via nuclear hormone receptors that directly regulate the transcription of target genes. EcR and E74 are key regulators in the ecdysone-inducible gene activation cascade in insects (Huet et al., 1995; Shirai et al., 2012). They are ‘early’ genes because they are directly induced by 20E as a first response. Their products activate the transcription of a few early-late genes, inactive their own transcription and further induce hundreds of late genes. The EcR/USP heterodimer regulates the expression of *EcR* and *E74* genes through binding to the ecdysone response element in their promoter region. The mRNA levels for *usp*, the other component of the ecdysone receptor heterodimer, remain essentially unchanged during the different developmental stages (Huet et al., 1995). This behaviour could be observed for these three genes in salivary glands of *C. riparius* triggered by ecdysone (Ozáez et al., 2014): In response to 20E exposure, the *EcR* gene was overexpressed earlier, *E74* expression was slightly retarded and there was no alteration in *usp* expression. Accordingly, these three genes together with the *ERR* gene have shown an ability to detect endocrine-disrupting activities in invertebrates and to define a putative different mode of actions for each EDC. The effect of short-term TBTO exposure on the expression of these four genes has been reported (Morales et al., 2013). To improve our knowledge about TBTO exposure and its putative molecular mechanism of toxicity, we examined the effects of long-term (96 h) TBTO exposure on *ERR*, *E74* and *usp* expression in *C. riparius* fourth instar larvae using real-time PCR (Fig. 3). Only exposure to 1 ng/L induced significant *usp* overexpression. *E74* and *ERR* expression was not altered in any studied condition. By contrast, *E74* and *ERR* genes are overexpressed after 24 h exposure (Morales et al., 2013). The ability of TBTO to induce *usp* expression shows a different behaviour compared with most other EDCs that have been tested in *C. riparius* (Planelló et al., 2008, 2011, 2015; Ozáez et al., 2013; Morales et al., 2014). Therefore, these data suggest that TBTO acts as an endocrine disruptor but does not mimic the hormone ecdysone because it can upregulate *EcR*, *E74*, *ERR* and *usp* after a 24 h exposure, while it only upregulates *EcR* and *usp* gene after 96 h. We observed similar behaviour only after short-term triclosan/5-chloro-2-(2,4-dichlorophenoxy) phenol exposure (Martínez-Paz et al., 2017). Although there is very little information about the endocrine disruptive effect of EE, NP and TBTO in invertebrates. Results obtained in Drosophila melanogaster showed that EE exposure revealed potential toxic and endocrine effects on adults of both sexes (Bovier et al., 2018). NP has been described as an endocrine disrupter in other insect species (Atli, 2013; Yuan et al., 2013) and in mollusks (Riva et al., 2010; Liu et al., 2011; De Lisa et al., 2013). The mechanism by which NP interferes with the hormonal system is unknown, but its effect as an endocrine disruptor is attributed to its molecular structure since it is like estradiol (Thiele et al., 1997). On the other hand, tributyltin synergizes with 20-hydroxyecdysone to activate transcription of the hormone-inducible genes in Daphnia sp. (Wang et al., 2011). Consequently, assessing the effect of EDCs on the transcription of all these four genes is interesting to reveal and classify different putative molecular mechanisms of toxicity.

### Bioaccumulation potential, bioavailability and physicochemical properties of EDCs: PCA and HCA analysis

We obtained several descriptors from *in silico* analysis to gather information about potential bioconcentration, bioavailability and a quantitative description of molecular structure for all the chemical compounds we explored in this work. The toxicity of EDCs should mostly be interpreted from the standpoint of its mode of action and the body burden in aquatic insects (Katagi and Tanaka, 2016). So that we might have a fuller picture of their effect, it is of interest to estimate the bioaccumulation potential and the bioavailability of the studied EDCs, as indicators of the putative body burden (Lai et al., 2002). The molecular lipophilicity – which is quantitively described by the octanol-water partition coefficient, represented by log *P* for neutral compounds – is one of the most important physicochemical properties that affects the bioavailability of compounds and it can be useful to estimate in vivo permeability. Hence, it can be used to understand bioaccumulation and exposure of wildlife to the environmental pollutant (Bhal, n.d.; Zhang et al., 2018).

**Fig. 4:**
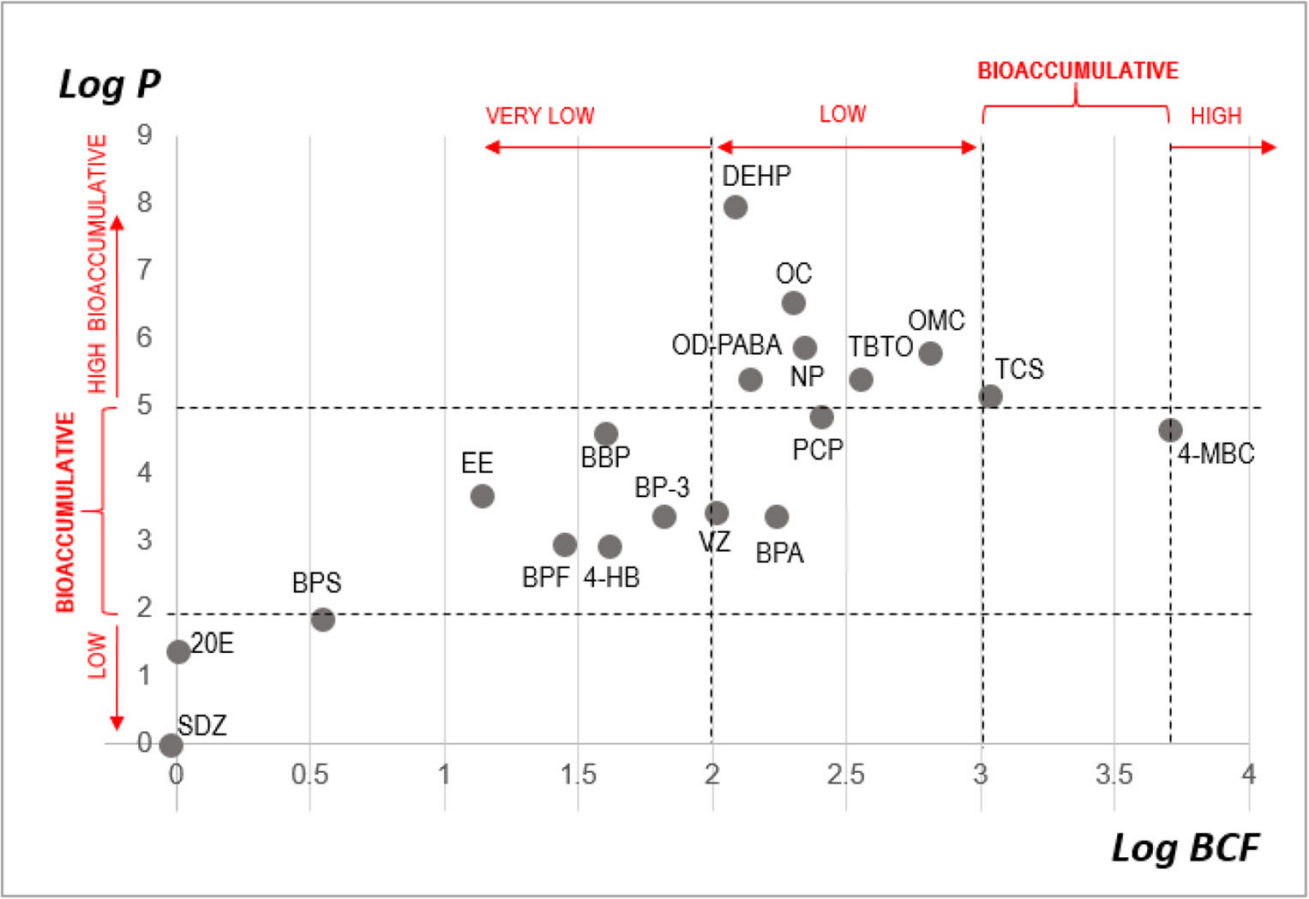
Bioaccumulation potential. Log bioconcentration factor (BCF, defined as the ratio of the amount of chemical in an aquatic organism to the amount of chemical in the water under conditions of equilibrium) versus miLogP, where *P* is the octanol-water partition coefficient. Both indicators have been calculated by Molinspiration (https://www.molinspiration.com)

Very lipophilic substances (log *P* > 5) are generally considered to have the greatest potential to bioaccumulate (without further consideration about metabolism or degradation), whereas substances with low lipophilicity (log *P* < 2) have relatively low bioaccumulation potential. On the other hand, the bioconcentration and bioaccumulation factors (BCF and BAF, respectively) are indicators that can be used to predict the bioaccumulation potential. It was proposed that there is not significantly bioaccumulation for BCF or BAF < 1000 and the bioaccumulation is high for BCF or BAF > 5000 (National Research Council, 2014). Thus, as can be seen in Fig. 4, very low bioaccumulation potential can be expected for SDZ and BPS, whereas, at the other end, 4-MCB would be highly bioaccumulative.

**Fig. 5:**
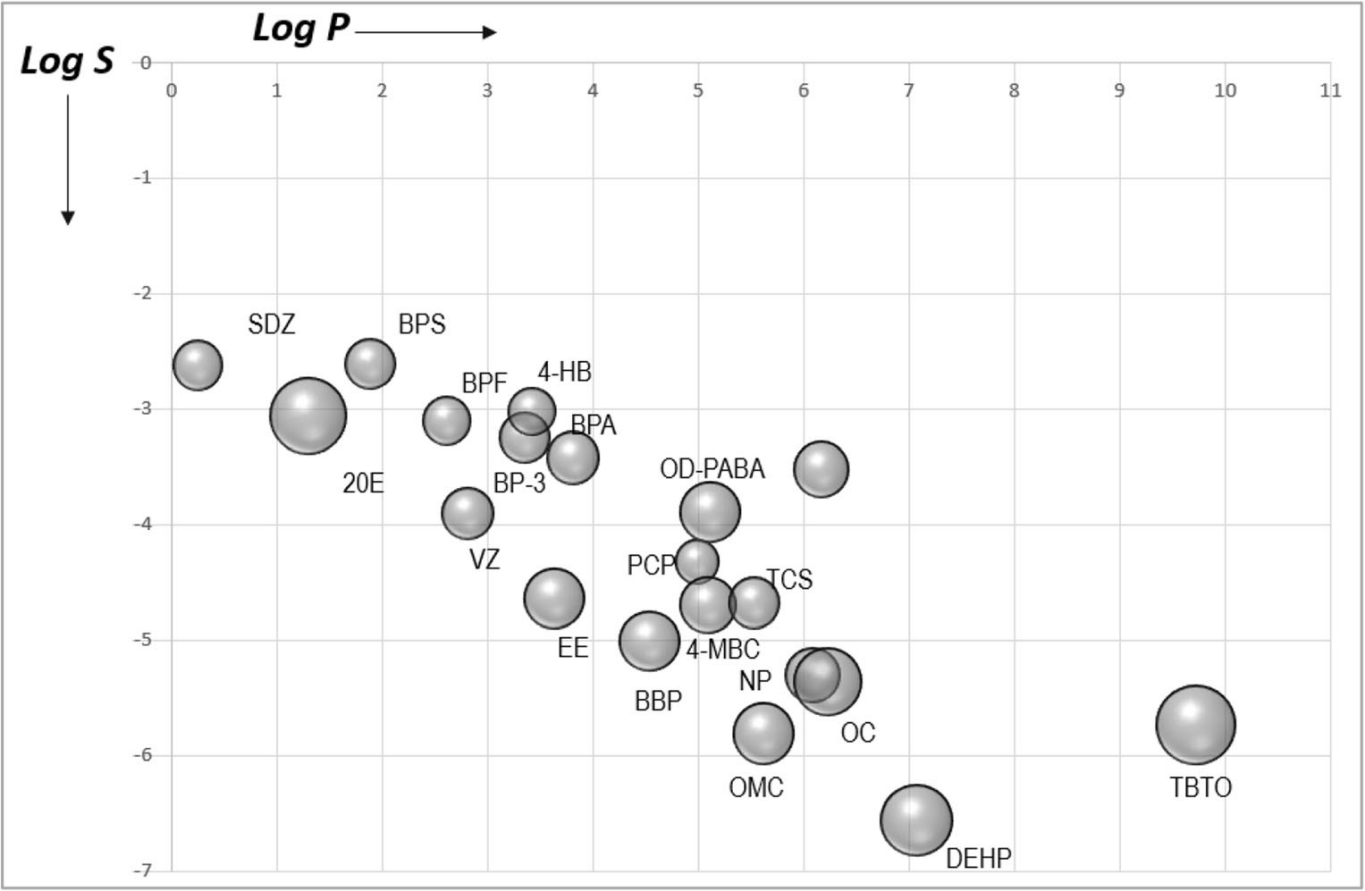
Ghose–Crippen octanol-water partition coefficient (log *P*) (lipophilicity) versus aqueous solubility (log *S*); both descriptors calculated by eDragon v1.0 (Tetko et al., 2005). The bubble size represents the molecular volume calculated by Molinspiration (https://www.molinspiration.com)

Furthermore, bioavailability is a measure of the amount of a chemical that can contact biological barriers and the rate at which it crosses and enters the organism to interfere tentatively in biological processes. While a lack of bioavailability is an indicator that the compound is likely to have low toxicity, high bioavailability does not suggest the compound is necessarily highly toxic. In aquatic species, bioavailability is positively correlated with the log *P* of the chemical; it is also influenced by aqueous solubility, molecular size and ionisation state. As molecular weight increases, aquatic bioavailability and toxicity generally decrease, where bioavailability is negligible at a molecular weight > 1000 amu and very poorly water-soluble chemicals (< 1 ppb) generally have low bioavailability and are less toxic (National Research Council, 2014). Figure 5 shows a putative estimation of the bioavailability, considering the log *P* (lipophilicity), log *S* (aqueous solubility) and molecular sizes for the analysed chemical compounds. Based on these results, the compounds with a bioavailability most similar to 20E are SDZ, BPS, VZ and BPF.

**Fig. 6:**
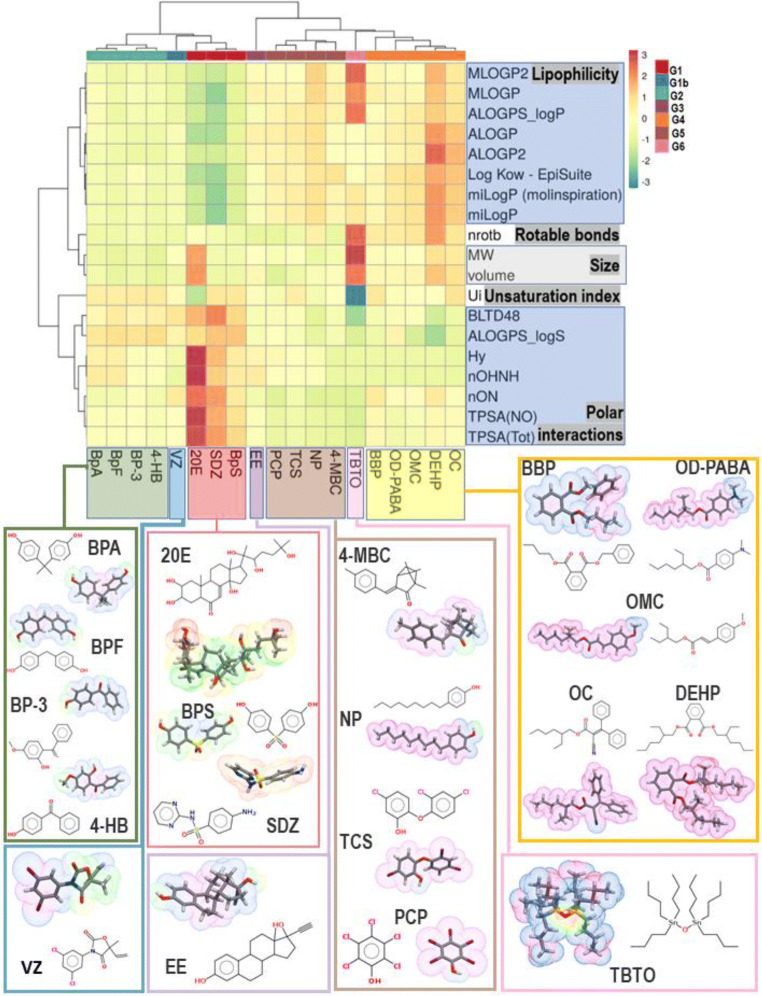
Heatmap showing colour codes for the value of the analysed descriptors. Rows are centred; unit variance scaling is applied to rows. Imputation is used to estimate missing values. Both rows and columns are clustered using correlation distance and average linkage. Molecular structures clustered in relation to their physicochemical properties showing molecular lipophilicity potential (MLP) on the molecular surface. Hydrophobic regions are highlighted by violet and blue, and hydrophilic regions are highlighted in orange and red. We calculated MLP from atomic hydrophobicity contributions with Molinspiration Cheminformatics free web services (https://www.molinspiration.com) and Molinspiration Galaxy 3D Structure Generator v2018.01_beta

Once inside cells, the biochemical behaviour and inherent toxicity of a chemical are dependent on its intrinsic physicochemical properties (National Research Council, 2014). The hydrophobicity, the polarity, the number of putative hydrogen bond acceptor/donor groups, the flexibility and the molecular size are all key attributes that determine the interaction between a chemical and its targets. Some molecular descriptors enable us to quantify these characteristics for each compound. Thus, we calculated selected descriptors by applying several methods (see Supplementary information): the molecular weight and volume, the topological polar surface area (TPSA), the octanol-water partition coefficient (*P*), the hydrogen bond acceptor/donor count, the hydrophilic factor, the aqueous solubility coefficient (log *S*), the number of rotatable bonds and the unsaturated index. To classify the chemical compounds based on these properties, we employed PCA and HCA. The results of this analysis are included in Supplementary information and are summarised in Fig. 6. Given that EDCs represent a broad class of molecules with divergent modes of action, this classification provides us with a convenient grouping to evaluate more easily their different effects on gene expression changes through their putative interaction with nuclear receptors. Consequently, we compiled the main previously reported experimental results showing the transcriptional activity changes of the ecdysone cascade genes *EcR*, *E74*, *usp* and *EER* in response to EDC exposure (Table 2); we sorted the gathered information according to this EDC classification.

**Table 2:** In vivo exposure effects of endocrine-disrupting chemicals on fourth instar larvae of *Chironomus riparius*

**Chemical**	***EcR***		***usp***		***E74***		***ERR***		**References**
	**Effect**	**Chemical exposure**	**Effect**	**Chemical exposure**	**Effect**	**Chemical exposure**	**Effect**	**Chemical exposure**	
**Group 1**									
**BPS**	↑	24 h ≥0.005 mg/L	N/A		↑	24 h 1 mg/L	↑	24 h ≥0.005 mg/L	Herrero et al., 2018; Morales et al., 2020
**SDZ**	↑	48 h 0.002 mg/L	N/A		↑	48 h 0.002 mg/L	N/A		Xie et al., 2019
**VZ**	↑	24 h 0.02 mg/L	N/A		**≡**	24 h 0.02 and 0.2 mg/L	N/A		Aquilino et al., 2016
	**≡**	48 h 0.02 and 0.2 mg/L			↑	48 h 0.02 mg/L			
**Group 2**									
**BPF**	↑	24 h ≥0.05 mg/L	N/A		↑	24 h 1 mg/L	N/A		Morales et al., 2020
**BPA**	↑	12 and 24 h ≥3 mg/L	N/A		**≡**	24 h ≥0.05	↑	24 and 96 h ≥0.005 mg/L	Morales et al., 2020; Park and Kwak, 2010; Planelló et al., 2008
**4-HB**	**≡**	24 h ≥0.1 mg/L	**≡**	24 h ≥0.1 mg/L	N/A		**≡**	24 h ≥0.1 mg/L	Ozáez et al., 2013
**BP-3**	**≡**	24 h ≥0.1 mg/L	↓	24 h 10 mg/L	N/A		**≡**	24 h ≥0.1 mg/L	Ozáez et al., 2013
**Group 3**									
**EE**	↑	24 h, 5 mg/L 96 h, 0.5 mg/L	N/A		N/A		N/A		This work
**Group 4**									
**OD-PABA**	↑	24 h 1 and 10 mg/L	**≡**	24 h ≥0.1 mg/L	N/A		**≡**	24 h ≥0.1 mg/L	Ozáez et al., 2013
**OMC**	↑	24 h 1 and 10 mg/L	**≡**	24 h ≥0.1 mg/L	N/A		**≡**	24 h ≥0.1 mg/L	Ozáez et al., 2013
**OC**	**≡**	24 h ≥0.1 mg/L	**≡**	24 h ≥0.1 mg/L	N/A		**≡**	24 h ≥0.1 mg/L	Ozáez et al., 2013
**BBP**	↑	24 h ≥0.1 mg/L	**≡**	24 h 0.01–1 mg/L (slight activation at ≥10 mg/L)	N/A		**≡**	24 h ≥10^-5^ mg/L (repression at 10^-6^ mg/L)	Herrero et al., 2015; Planelló et al., 2011
	↓	48 h ≥10^-6^ mg/L					↓	48 h 10^-6^ mg/L	
**DEHP**	↓	24 h 100 mg/L	**≡**	24 h 0.01–100 mg/L	N/A		↑	24 h 50 mg/L	Herrero et al., 2017; Park and Kwak, 2010; Planelló et al., 2011
	↓	48, 72 and 96 h ≥10^-6^ mg/L					↑	96 h ≥0.5 mg	
**Group 5**									
**NP**	↑	12 and 24 h, 0.05 mg/L; 48 and 72 h, 0.01 mg/L	N/A		N/A		↑	24 and 96 h ≥0.001 mg/L	Nair and Choi, 2012; Park and Kwak, 2010; this work
	↓	24 h 0.1 mg/L					↓	24 h 0.1 mg/L	
**4-MBC**	↑	24 h ≥0.1 mg/L	**≡**	24 h ≥0.1 mg/L	N/A		**≡**	24 h ≥0.1 mg/L	Ozáez et al., 2013
**PCP**	**≡**	12 and 24 h 0.250 mg/L	**≡**	12, 24 and 96 h ≥0.025 mg/L					Morales et al., 2014
	↑	96 h 0.250 mg/L			↑	96 h ≥0.025 mg/L	↑	96 h ≥0.025 mg/L	
**TCS**	↑	24 h 1 mg/L	↑	24 h 0.010 mg/L	↑	24 h 0.010 mg/L	↑	24 h 0.010 mg/L	Martínez-Paz et al., 2017
**Group 6**									
**TBTO**	↑	24 h 1×10^-6^ mg/L	↑	24 h 1×10^-6^ mg/L	↑	24 h 1×10^-6^ mg/L	↑	24 h 1×10^-5^ mg/L	Morales et al., 2013
	↑	96 h 1×10^-6^ and 1×10^-5^ mg/L	↑	96 h ≥1×10^-7^ mg/L	**≡**	96 h ≥1×10^-7^ mg/L	**≡**	96 h ≥1×10^-7^ mg/L	This work

*Symbols***: ≡**, no significant effect; ↑, overexpression; ↓, downregulated; N/A, not available

*20E*, 20-hydroxyecdysone; *EE*, ethinyl oestradiol; *PCP*, pentachlorophenol; *TCS*, triclosan/5-chloro-2-(2,4-dichlorophenoxy)phenol; *NP*, p-nonylphenol; *BPS*, bisphenol S; *BPA*, bisphenol A; *BPF*, bisphenol F; *BP-3*, benzophenone-3; *4-HB*, 4-hidroxybenzophenone; *DEHP*, di(2-ethylhexyl) phthalate; *BBP*, butyl benzyl phthalate; *OMC*, octyl-p-methoxycinnamate; *OC*, octocrylene; *VZ*, vinclozolin; *SDZ*, sulfadiazine; *4-MBC*, 4-methylbenzylidene camphor; *OD-PABA*, octyl dimethyl-p-aminobenzoate; *TBT*, tributyltin; *TBTO*, bis(tributyltin) oxide

We differentiated six groups based on the physicochemical properties of the EDCs (considering SDZ, BpS and VZ in one unique group, marked as G1 and G1b in Fig. 6). Using this approach, we tried to simplify the analysis to get information that, combined with experimental data, could provide a useful classification of EDCs in advance and help to anticipate the putative results that can be obtained by other compounds. Thus, regarding these physicochemical properties, compounds more similar to 20E are those in group 1. Accordingly, it would be expected that they could mimic the 20E effect, just as it was observed in the experiments with VZ (Aquilino et al., 2016), where the sequential activation of *EcR* and *E74* genes reproduces a similar response to the natural ecdysteroid (see Table 2). Responses to BPS and SDZ are compatible with these facts, but more experiments with longer exposure times are necessary for conformation. For all these compounds, the expected bioaccumulation potential and bioavailability are low (Figs. 4 and 5, as it was experimentally shown for SDZ (Xie et al., 2019) but, once inside the cell, they could interact with the EcR/USP heterodimer in a similar way to 20E. We found a similar trend for BPF, but, on the contrary, there were not significant effects for exposure to 4-hidroxybenzophenone and BP-3, or they were observed at higher doses, as it is the case for bisphenol A. These compounds belong to group 2, where the expected bioaccumulation potential is increasing, but they are less similar to 20E in their properties. In group 4, there is a change in the trend, with *EcR* being upregulated after octyl dimethyl-p-aminobenzoate and octyl-p-methoxycinnamate exposure and downregulated after BBP and DEHP exposure. Bioaccumulation potential for compounds in these groups are higher and are particularly great for DEHP. This phenomenon could be one of the causes of their effect as endocrine disruptors, but their physicochemical differences relative to 20E could be responsible for other mode(s) of action. Similarly, great bioavailability and bioaccumulation potential is expected for TBTO, but its chemical structure and properties could provide a basis for a different molecular mechanism of toxicity, as the upregulation of the *usp* gene indicate.

## Conclusion

In summary, the ability of different chemical compounds to activate/repress transcription of the evaluated ecdysone cascade genes can be related to their endocrine disruptive effects and reveal different underlying molecular mechanisms of toxicity. Being able to classify chemicals based on the effects they have at the molecular level helps to develop rationale, cost-effective and comprehensive testing approaches to evaluate environmental hazards. A previous classification of EDCs based on their physicochemical properties seems to be of interest to understand those effects. For any future work, it should be preferable to assess the effect of short-term (24 h) and long-term (96 h) treatment and three different sub-lethal dosages for each considered EDCs to identify the different patterns of changes on the *EcR* transcription and to get a more complete view.

## Data Availability

All data generated or analyzed during this study are included in this published article
